# Supplementary material for: Scleral PERK and ATF6 as targets of myopic axial elongation of mouse eyes
Source: Nat Commun. 2022 Oct 10;13:5859. doi: 10.1038/s41467-022-33605-1 (PMC9550863; doi:10.1038/s41467-022-33605-1)
Supplement: Supplementary file 1 — Supplementary Information [file 41467_2022_33605_MOESM1_ESM.pdf]

Supplementary Information for:

Scleral PERK and ATF6 as targets of myopic axial elongation of mouse eyes

Shin-ichi Ikeda[1,2], Toshihide Kurihara[1,2*], Xiaoyan Jiang[1,2], Yukihiro Miwa[1,2], Deokho Lee[1,2], Naho Serizawa[1,2], Heonuk Jeong[1,2], Kiwako Mori[1,2], Yusaku Katada[1,2], Hiromitsu Kunimi[1,2], Nobuhiro Ozawa[1,2], Chiho Shoda[1,2], Mari Ibuki[1,2], Kazuno Negishi[2], Hidemasa Torii[1,2], Kazuo Tsubota[2,3*]

[1] Laboratory of Photobiology, Keio University School of Medicine, 35 Shinanomachi, Shinjuku-ku, Tokyo 160-8582, Japan

[2] Department of Ophthalmology, Keio University School of Medicine, 35 Shinanomachi, Shinjuku-ku, Tokyo 160-8582, Japan

[3] Tsubota Laboratory, Inc., 34 Shinanomachi, Shinjuku-ku, Tokyo 160-0016 Japan

[*]E-mail: kurihara@z8.keio.jp / tsubota@tsubota-lab.com

Supplemental Figure 1-10
Supplemental Table 1-8

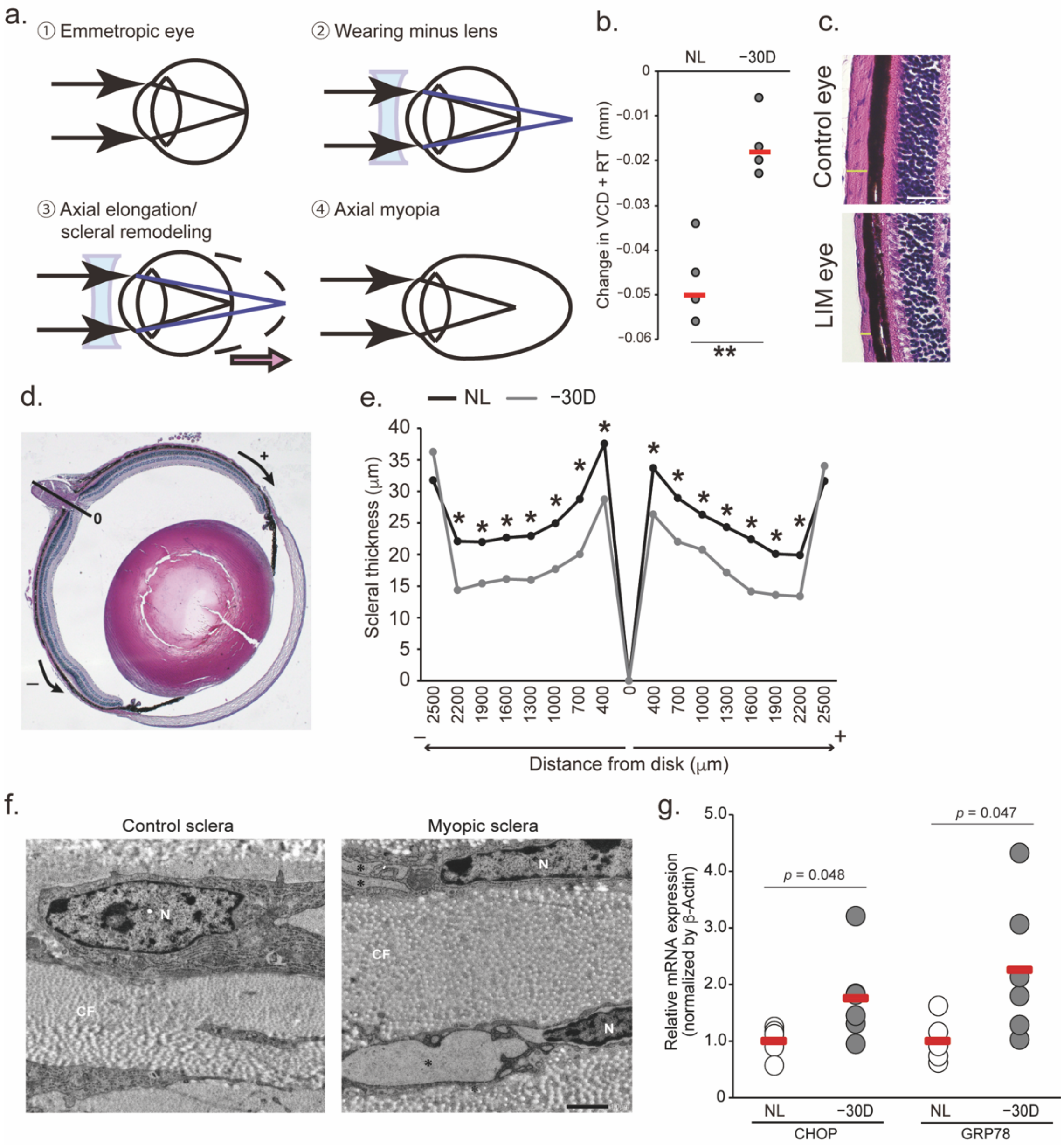
a.
① Emmetropic eye
② Wearing minus lens
③ Axial elongation/ scleral remodeling
④ Axial myopia
b.
NL
−30D
Change in VCD + RT (mm)
0
-0.01
-0.02
-0.03
-0.04
-0.05
-0.06
**
c.
Control eye
LIM eye
d.
0
+
−
e.
NL
−30D
Scleral thickness (μm)
Distance from disk (μm)
f.
Control sclera
Myopic sclera
N
CF
g.
Relative mRNA expression (normalized by β-Actin)
p = 0.048
p = 0.047
NL
−30D
CHOP
GRP78

Supplemental Figure 1 | Lens-induced myopia in mice and chicks.

(a) Schematic structure of lens-induced myopia (LIM). **(b)** Changes in vitreous chamber depth (VCD) + retinal thickness (RT) during 3-week lens-induced myopia (LIM) in C57BL6J mice (n = 4). NL: No Lens control, -30D: minus 30 D lens-wearing eye. ** p = 0.002; Student's two-tailed t-test. **(c)** Haematoxylin and eosin staining of control and LIM eyes. Yellow bars indicate scleral thickness (n = 5 per group); scale bar, 50 μm. **(d)** Measurement of scleral thickness. The position of the optic disk is represented by '0', and superior (+) and inferior (-) distances from disk were measured. At 400, 700, 1000, 1300, 1600, 1900, 2200 and 2500 μm, scleral thickness was measured. **(e)** Scleral thickness of control (black line) and LIM (grey line) eyes measured in HE-stained sections (n = 5 per group); * $p < 0.05$; Student's two-tailed t-test (The exact p-values are, from left to right: 0.019, 0.002, 0.015, 0.001, 0.003, 0.004, 0.004, 0.006, 0.011, 0.015, 0.00063, 0.00045, 0.0003, 0.003, 0.026). **(f)** TEM images of control and LIM sclerae of White Leghorn chicks (n = 3 per group). Scale bars, 2 μm. **(g)** mRNA expression levels of CHOP and GRP78 in control and LIM sclerae of White Leghorn chicks (n = 6 per group). NL group was assigned a value of 1.0. The p values were determined by Student's two-tailed t-test. Source data are provided as a Source Data file.

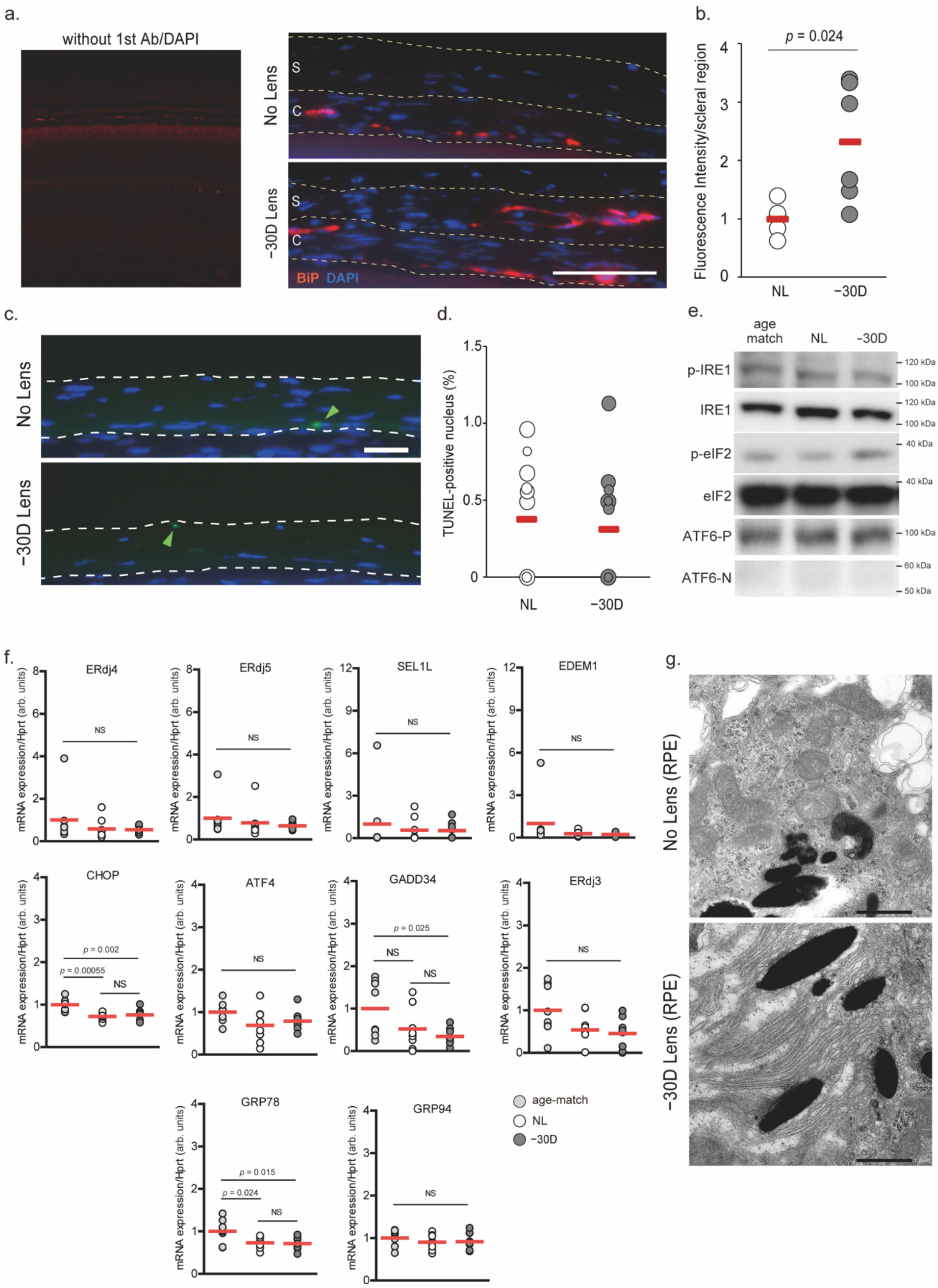
a.
without 1st Ab/DAPI
No Lens
-30D Lens
S
C
BiP DAPI
b.
p = 0.024
Fluorescence Intensity/scleral region
NL
-30D
c.
No Lens
-30D Lens
d.
TUNEL-positive nucleus (%)
NL
-30D
e.
age match
NL
-30D
p-IRE1
IRE1
p-eIF2
eIF2
ATF6-P
ATF6-N
120 kDa
100 kDa
40 kDa
60 kDa
50 kDa
f.
ERdj4
ERdj5
SEL1L
EDEM1
CHOP
ATF4
GADD34
ERdj3
GRP78
GRP94
mRNA expression/Hprt (arb. units)
NS
p = 0.002
p = 0.00055
p = 0.025
p = 0.015
p = 0.024
age-match
NL
-30D
g.
No Lens (RPE)
-30D Lens (RPE)

Supplemental Figure 2 | Ophthalmological and cellular changes after LIM in the sclera and the retina.

(a) Immunohistochemical images stained for Bip (red) and DAPI (nucleus, blue) of no lens (middle panel) and minus 30 D lens-wearing (right panel) sclerae in C57BL6J mice. The area between the yellow dot lines is the sclera (S), and the area between the yellow and green dot lines is the choroid (C). The images were acquired from posterior region (in the area around 400-1000 μm from the disc). The left panel showed the image of no-primary antibody control. Showing representative images in four biologically independent samples; scale bar, 20 μm. **(b)** Bar graph showing the results of the quantification of the fluorescence intensity in Supplemental Fig 2a (n = 5 in NL group, n = 6 in -30 D group). NL group was assigned a value of 1.0. The p value was determined by Student's two-tailed t-test. **(c)** TUNEL images (green: TUNEL, blue: DAPI) of control and LIM sclerae in C57BL6J mice. The bottom part facing towards the choroid. The area between the yellow dot lines is the sclera. The images were acquired from posterior region (in the area around 400-1000 μm from the disc). Upper panel: no lens control, lower panel: LIM. Arrowheads indicate TUNEL-positive nuclei. Showing representative images in four biologically independent samples.; scale bar, 100 μm. **(d)** Bar graph showing the ratio of TUNEL-positive nuclei to DAPI-positive nuclei in sclera. The p value was determined by Student's two-tailed t-test. Three images were acquired from each of the four slides (12 images in total) for quantitative analysis. **(e)** IRE1, eIF2 and ATF6 did not activated by LIM in retina determined by Western blotting. Showing representative blots in four biologically independent samples. **(f)** UPR target gene expression level determined by quantitative PCR in age matched control (right grey column), No-Lens control (white column) and LIM (grey column) retina for 3-week LIM

(n = 8). Age-match group was assigned a value of 1.0. The p values were determined by ANOVA with Bonferroni's Multiple Comparison Test. **(g)** TEM images of retinal pigment epithelium from control and myopia-induced eyes. (n = 3 per group). Scale bars, 1 μm. Source data are provided as a Source Data file.

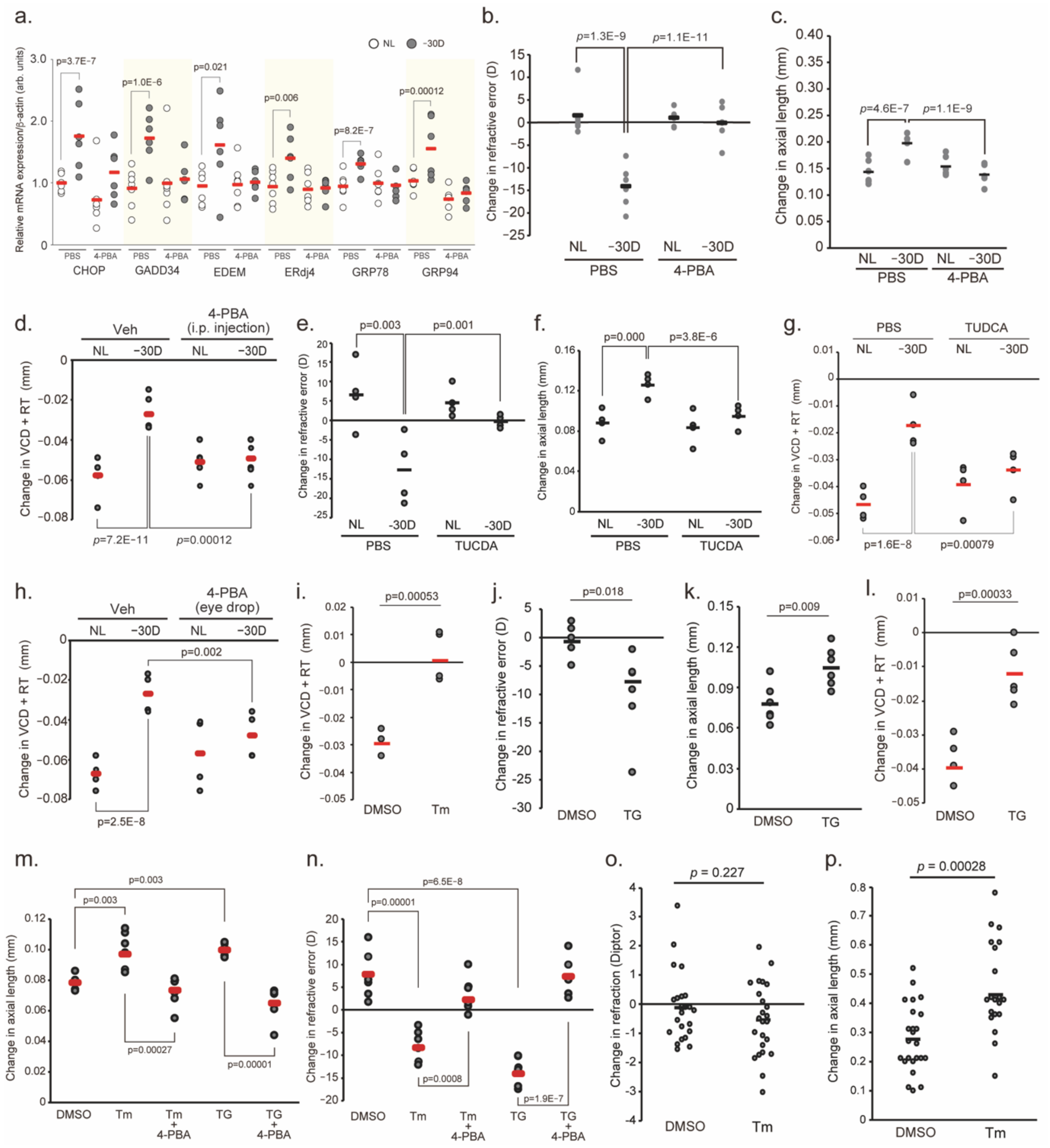
a.
NL
-30D
Relative mRNA expression/β-actin (arb. units)
p=3.7E-7
p=1.0E-6
p=0.021
p=0.006
p=8.2E-7
p=0.00012
PBS
4-PBA
CHOP
GADD34
EDEM
ERdj4
GRP78
GRP94
b.
p=1.3E-9
p=1.1E-11
Change in refractive error (D)
NL
-30D
PBS
4-PBA
c.
p=4.6E-7
p=1.1E-9
Change in axial length (mm)
d.
Veh
4-PBA
(i.p. injection)
Change in VCD + RT (mm)
p=7.2E-11
p=0.00012
e.
p=0.003
p=0.001
TUCDA
f.
p=0.000
p=3.8E-6
g.
TUDCA
p=1.6E-8
p=0.00079
h.
4-PBA
(eye drop)
p=0.002
p=2.5E-8
i.
p=0.00053
DMSO
Tm
j.
p=0.018
TG
k.
p=0.009
l.
p=0.00033
m.
p=0.003
p=0.003
p=0.00027
p=0.00001
Tm + 4-PBA
TG + 4-PBA
n.
p=6.5E-8
p=0.00001
p=0.0008
p=1.9E-7
o.
p = 0.227
Change in refraction (Diptor)
p.
p = 0.00028

Supplemental Figure 3 | Intervention of scleral ER stress affects eyeball size and refraction in mice and chicks.

(a) Intraperitoneal (i.p.) injection of 4-phenylbutyric acid (4-PBA; 200 mg/kg/day) suppresses 3-week-LIM-induced increase in UPR gene expression determined by quantitative PCR in control (No Lens, white column) and LIM (-30 D, grey column) sclerae (n = 6 per group). The p values were determined by two-tailed Generalized Estimating Equations. 0.00012**(b)** LIM-induced myopic shift in refraction was inhibited by 4-PBA administration for 1 week (n = 6 per group). The p values were determined by Generalized Estimating Equations. **(c)** LIM-induced axial elongation was inhibited by 4-PBA administration for 1 week (n = 6 per group). The p values were determined by Generalized Estimating Equations. **(d)** LIM-induced attenuation of vitreous chamber depth (VCD) + retinal thickness (RT) shortening suppressed by 4-PBA administration for 3 week (n = 6 per group). The p values were determined by Generalized Estimating Equations. **(e)** LIM-induced myopic shift in refraction was inhibited by TUDCA administration for 3 weeks (n = 4). The p values were determined by two-tailed Generalized Estimating Equations. **(f)** LIM-induced axial elongation was inhibited by TUDCA administration for 3 weeks (n = 4 per group). The p values were determined by two-tailed Generalized Estimating Equations. **(g)** LIM-induced attenuation of VCD + RT shortening suppressed by TUDCA administration for 3 week (n = 4 per group). The p values were determined by two-tailed Generalized Estimating Equations. **(h)** LIM-induced attenuation of VCD + RT shortening suppressed by 2% 4-PBA instillation for 3 week (n = 6 per group). The p values were determined by two-tailed Generalized Estimating Equations. **(i)** Single tunicamycin (Tm, 50 μg/mL) instillation suppressed VCD + RT shortening during eye growth (n = 5). The p values were determined by

Student's two-tailed t-test. **(j)** Single thapsigargin (TG: 10 μM) instillation induced refractive error (n = 6). The p values were determined by Student's two-tailed t-test. **(k)** Single TG (10 μM) instillation induced pathological axial elongation (n = 6 per group). The p values were determined by Student's two-tailed t-test. **(l)** Single TG (10 μM) instillation suppressed VCD + RT shortening during eye growth (n = 5). The p values were determined by Student's two-tailed t-test. **(m)** 4-PBA suppressed single Tm or TG instillation-induced axial elongation. The p values were determined by one-way ANOVA with Tukey HSD. **(n)** 4-PBA suppressed single Tm or TG instillation-induced refractive error. The p values were determined by one-way ANOVA with Tukey HSD. **(o)** Effect of single tunicamycin (Tm; 50 μg/mL) instillation on refraction in white Leghorn chicks. Both groups included 12 chicks (24 eyes). The p value was determined by Student's two-tailed t-test. **(p)** Effect of single Tm (50 μg/mL) instillation on axial elongation for 1 week in white Leghorn chicks. Both groups included 12 chicks (24 eyes). The p values were determined by Student's two-tailed t-test. Source data are provided as a Source Data file.

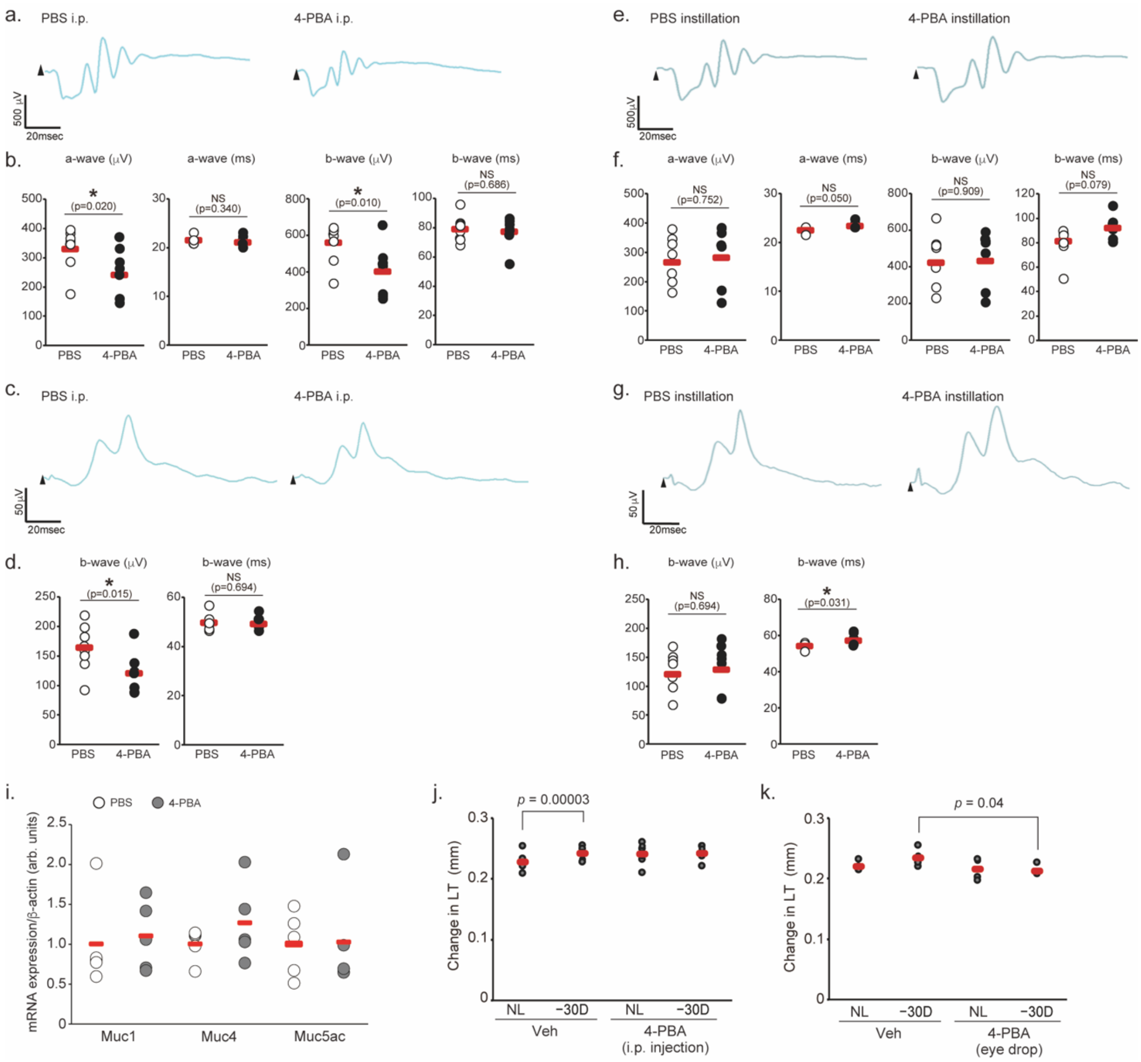
a.
PBS i.p.
4-PBA i.p.
500 µV
20msec
b.
a-wave (µV)
a-wave (ms)
b-wave (µV)
b-wave (ms)
(p=0.020)
(p=0.340)
(p=0.010)
(p=0.686)
NS
PBS
4-PBA
c.
PBS i.p.
4-PBA i.p.
50 µV
20msec
d.
b-wave (µV)
b-wave (ms)
(p=0.015)
(p=0.694)
e.
PBS instillation
4-PBA instillation
500µV
20msec
f.
a-wave (µV)
a-wave (ms)
b-wave (µV)
b-wave (ms)
(p=0.752)
(p=0.050)
(p=0.909)
(p=0.079)
g.
PBS instillation
4-PBA instillation
50µV
20msec
h.
b-wave (µV)
b-wave (ms)
(p=0.694)
(p=0.031)
i.
PBS
4-PBA
mRNA expression/β-actin (arb. units)
Muc1
Muc4
Muc5ac
j.
p = 0.00003
Change in LT (mm)
NL
−30D
Veh
4-PBA
(i.p. injection)
k.
p = 0.04
Change in LT (mm)
NL
−30D
Veh
4-PBA
(eye drop)

Supplemental Figure 4 | Side effect of 4-PBA administration in another component of the eye.

(a) Representative ERG waveforms (50 cd・s/m2) of scotopic condition in PBS or 4-PBA i. p. injected mice. **(b)** Averaged amplitudes (left panels) and implicit time (right panels) of a-wave and b-wave in scotopic condition of PBS or 4-PBA i. p. injected mice (n = 9, respectively). The p values were determined by Student's two-tailed t-test. **(c)** Representative ERG waveforms (20 cd・s/m2) of photopic condition in PBS or 4-PBA i. p. injected mice. **(d)** Averaged amplitudes (left panels) and implicit time (right panels) of b-wave in photopic condition of PBS or 4-PBA i. p. injected mice (n = 9, respectively). The p values were determined by Student's two-tailed t-test. **(e)** Representative ERG waveforms of scotopic condition in PBS or 4-PBA instilled mice. **(f)** Averaged amplitudes (left panels) and implicit time (right panels) of a-wave and b-wave in scotopic condition of PBS or 4-PBA instilled mice (n = 8, respectively). The p values were determined by Student's two-tailed t-test. **(g)** Representative ERG waveforms of photopic condition in PBS or 4-PBA instilled mice. **(h)** Averaged amplitudes (left panels) and implicit time (right panels) of b-wave in photopic condition of PBS or 4-PBA instilled mice (n = 7, respectively). The p values were determined by Student's two-tailed t-test. **(i)** Gene expression level of Mucin 1, 4 and 5ac in conjunctiva after 3 weeks of ocular administration of PBS (white column) and 4-PBA (gray column). Student's two-tailed t-test showed no significant differences. **(j)** Changes in lens thickness (LT) for 3 weeks LIM with PBS or 4-PBA i. p. injection. The p value was determined by two-tailed Generalized Estimating Equations. **(k)** Changes in lens thickness (LT) for 3 weeks LIM with PBS or 4-PBA instillation. The p value was determined by two-tailed Generalized Estimating Equations. Source data are provided as a Source Data file.

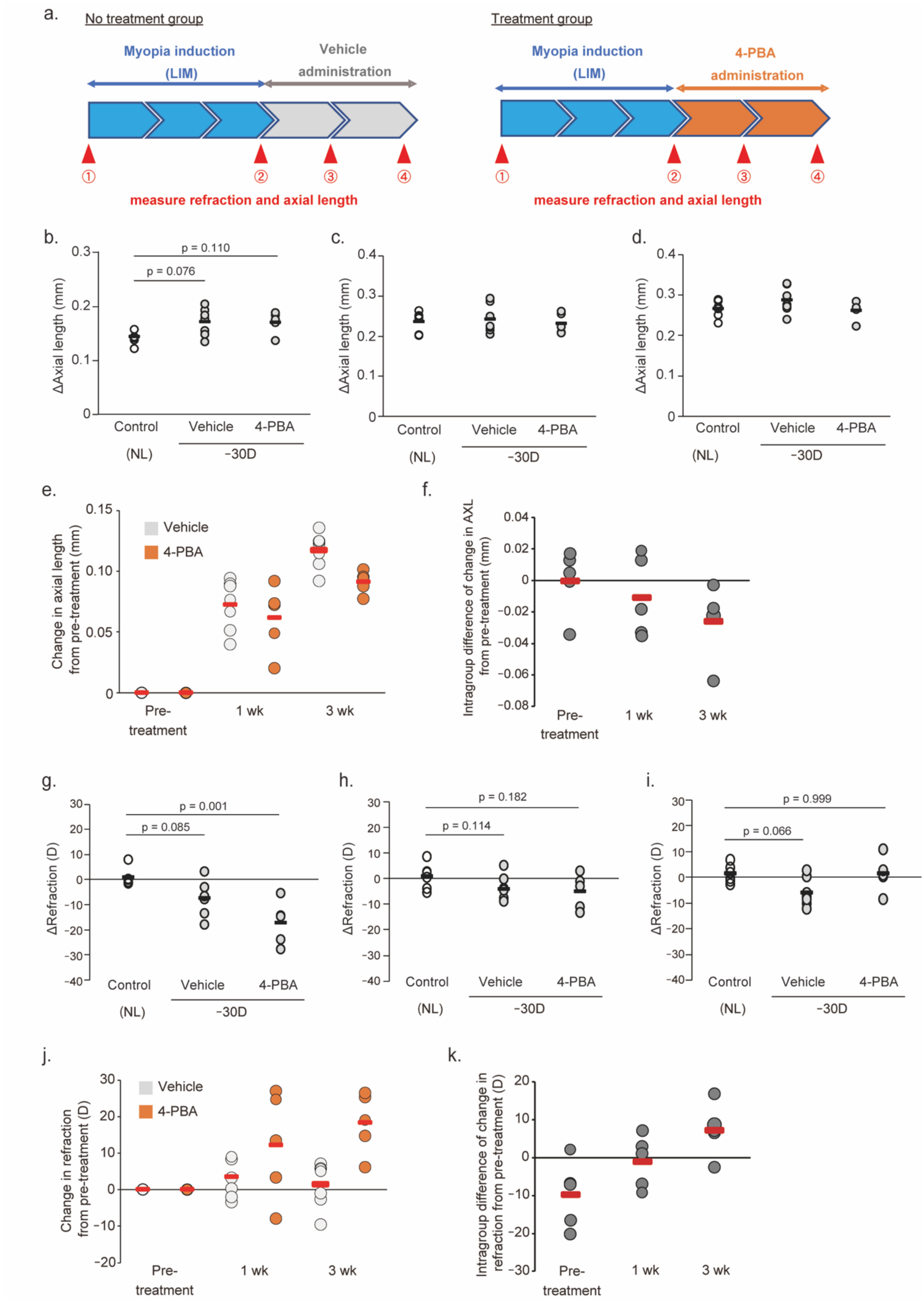
a.
No treatment group
Myopia induction (LIM)
Vehicle administration
① ② ③ ④
measure refraction and axial length
Treatment group
Myopia induction (LIM)
4-PBA administration
① ② ③ ④
measure refraction and axial length
b.
p = 0.110
p = 0.076
ΔAxial length (mm)
Control (NL)
Vehicle
4-PBA
-30D
c.
ΔAxial length (mm)
Control (NL)
Vehicle
4-PBA
-30D
d.
ΔAxial length (mm)
Control (NL)
Vehicle
4-PBA
-30D
e.
Vehicle
4-PBA
Change in axial length from pre-treatment (mm)
Pre-treatment
1 wk
3 wk
f.
Intragroup difference of change in AXL from pre-treatment (mm)
Pre-treatment
1 wk
3 wk
g.
p = 0.001
p = 0.085
ΔRefraction (D)
Control (NL)
Vehicle
4-PBA
-30D
h.
p = 0.182
p = 0.114
ΔRefraction (D)
Control (NL)
Vehicle
4-PBA
-30D
i.
p = 0.999
p = 0.066
ΔRefraction (D)
Control (NL)
Vehicle
4-PBA
-30D
j.
Vehicle
4-PBA
Change in refraction from pre-treatment (D)
Pre-treatment
1 wk
3 wk
k.
Intragroup difference of change in refraction from pre-treatment (D)
Pre-treatment
1 wk
3 wk

Supplemental Figure 5 | 4-PBA instillation is effective for myopia prevention and treatment.

(a) Experimental design of the experiments. After 3 weeks of LIM (without any treatment such as 4-PBA), the minus lenses and frames were removed, and PBS or 4-PBA eye drops were administered for 3 weeks. **(b)** Axial elongation during the lens-induced myopia (LIM) period (② - ①) in the control (n = 6), vehicle (n = 7), and 4-PBA (n = 5)-instilled groups. The p values were determined by one-way ANOVA with Tukey HSD. **(c, d)** Axial elongation for **(c)** 1-week treatment (③ - ①) and **(d)** 3-week treatment (④ - ①) in the control (n = 6), vehicle (n = 7), and 4-PBA (n = 5)-instilled groups. **(e)** Axial elongation from pretreatment (②) to 1-week (③ - ②) or 3-week (④ - ②) treatment in the vehicle (n = 7) and 4-PBA (n = 5)-instilled groups. **(f)** Differences between axial length of each 4-PBA-treated mouse and the average axial length of the vehicle-treated group (n = 5). **(g)** Refractive error during the LIM period (② - ①) in the control (n = 6), vehicle (n = 7), and 4-PBA (n = 5)-instilled groups. The p values were determined by one-way ANOVA with Tukey HSD. **(h, i)** Refractive error for **(h)** 1-week treatment (③ - ①) and **(i)** 3-week treatment (④ - ①) in the control (n = 6), vehicle (n = 7), and 4-PBA (n = 5)-instilled groups. The p values were determined by one-way ANOVA with Tukey HSD. **(j)** Refractive error from pretreatment (②) to 1-week (③ - ②) or 3-week (④ - ②) treatment in the vehicle (n = 7) and 4-PBA (n = 5)-instilled groups. **(k)** Differences between refraction of each 4-PBA treated mouse and the average refraction of the vehicle-treated group (n = 5). Source data are provided as a Source Data file.

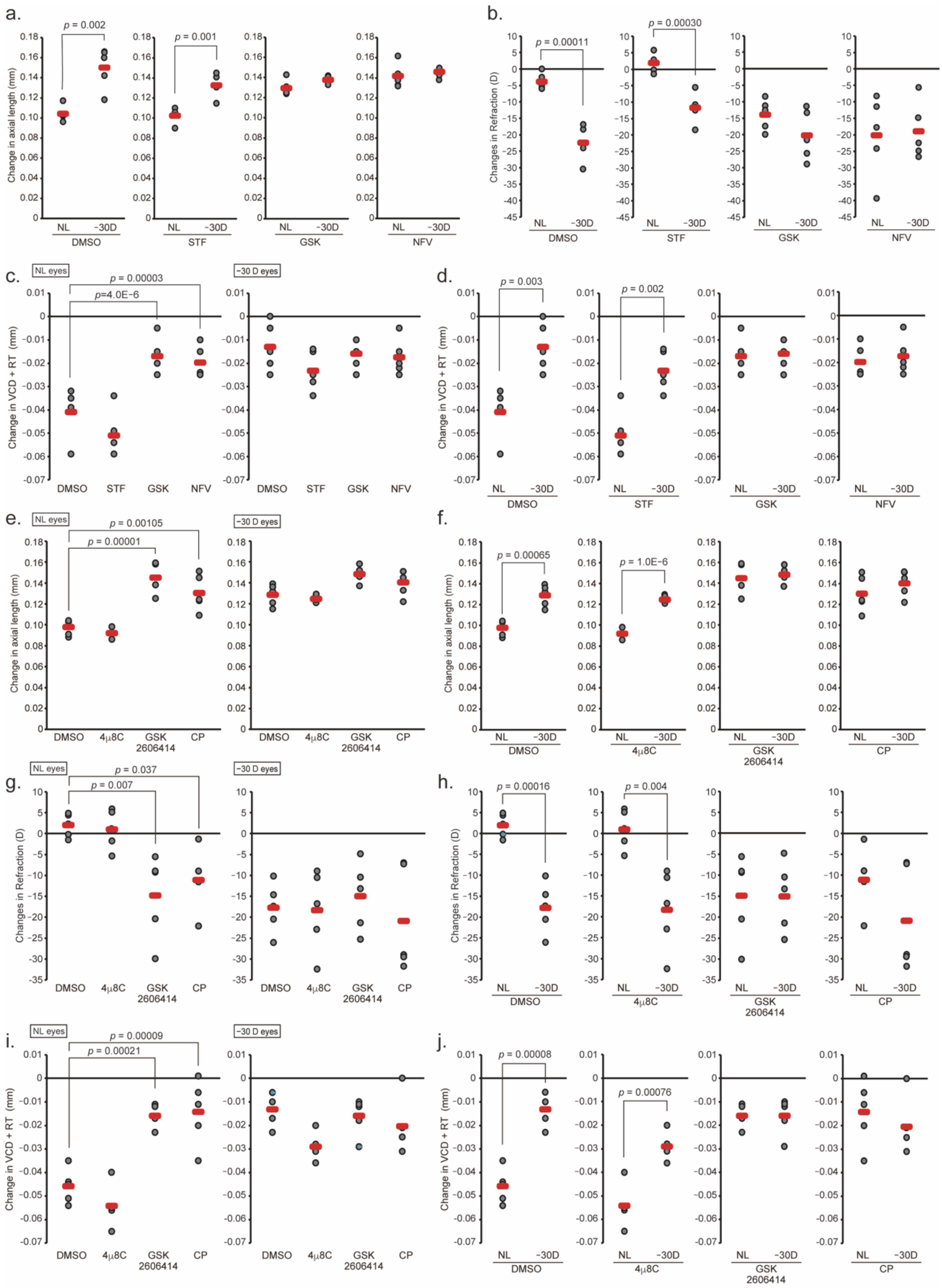
a.
p = 0.002
p = 0.001
Change in axial length (mm)
NL -30D
DMSO
STF
GSK
NFV
b.
p = 0.00011
p = 0.00030
Changes in Refraction (D)
c.
NL eyes
-30 D eyes
p = 0.00003
p=4.0E-6
Change in VCD + RT (mm)
DMSO STF GSK NFV
d.
p = 0.003
p = 0.002
e.
p = 0.00105
p = 0.00001
DMSO 4μ8C GSK 2606414 CP
f.
p = 0.00065
p = 1.0E-6
g.
p = 0.037
p = 0.007
h.
p = 0.00016
p = 0.004
i.
p = 0.00009
p = 0.00021
j.
p = 0.00008
p = 0.00076

Supplemental Figure 6 | Effect of single UPR inhibitor instillation on myopia development.

(a) Comparison of axial elongation between non-lens-wearing eyes (NL) and minus-lens-wearing eyes (-30D) in DMSO (as a control), STF080310 (STF; 100 μM), GSK2656157 (GSK; 100 μM), and nelfinavir (NFV; 100 μM)-administrated mouse. The p values were determined by Student's two-tailed t-test. **(b)** Comparison of refraction between NL and -30D eyes in DMSO, STF GSK and NFV-administrated mouse. The p values were determined by Student's two-tailed t-test. **(c)** Effects of STF, GSK, and NFV eye drops on VCD+RT in NL and -30 D eyes (n = 5 per group). The p values were determined by two-tailed Generalized Estimating Equations. **(d)** Comparison of VCD+RT between NL and -30D eyes in DMSO, STF GSK and NFV-administrated mouse. The p values were determined by Student's two-tailed t-test. **(e)** Effects of 4μ8C, GSK2606414, and Ceapin A-7 instillation (100 μM, respectively) eye drops on axial elongation in NL and -30 D eyes (n = 5 per group). The p values were determined by one-way ANOVA with Tukey HSD. **(f)** Comparison of axial elongation between NL and -30D eyes in DMSO, 4μ8C, GSK2606414, and Ceapin A-7-administrated mouse. The p values were determined by Student's two-tailed t-test. **(g)** Effects of 4μ8C, GSK2606414, and Ceapin A-7 instillation eye drops on refractive error in NL and -30 D eyes (n = 5 per group). The p values were determined by one-way ANOVA with Tukey HSD. **(h)** Comparison of refractive error between NL and -30D eyes in DMSO, 4μ8C, GSK2606414, and Ceapin A-7-administrated mouse. The p values were determined by Student's two-tailed t-test. **(i)** Effects of 4μ8C, GSK2606414, and Ceapin A-7 instillation eye drops on VCD+RT in NL and -30 D eyes (n = 5 per group). The p values were determined by one-way ANOVA with Tukey HSD. **(j)** Comparison of VCD+RT between NL and -30D eyes in DMSO,

4μ8C, GSK2606414, and Ceapin A-7-administrated mouse. The p values were determined by Student's two-tailed t-test. Source data are provided as a Source Data file.

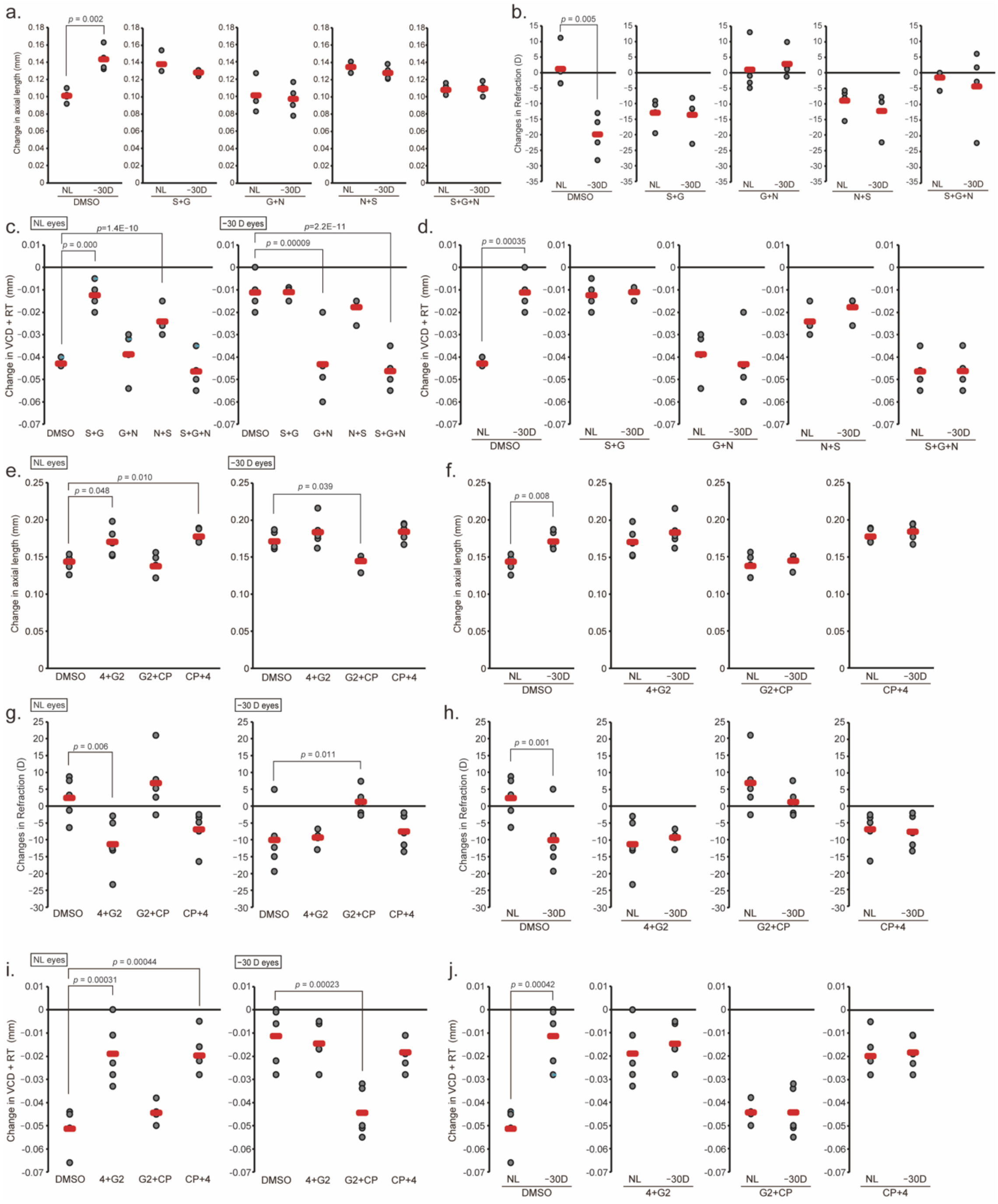

a.
p = 0.002
Change in axial length (mm)
NL -30D
DMSO
S+G
G+N
N+S
S+G+N
b.
p = 0.005
Changes in Refraction (D)
NL -30D
DMSO
S+G
G+N
N+S
S+G+N
c.
NL eyes
p=1.4E-10
p = 0.000
-30 D eyes
p=2.2E-11
p = 0.00009
Change in VCD + RT (mm)
DMSO S+G G+N N+S S+G+N
d.
p = 0.00035
Change in VCD + RT (mm)
NL -30D
DMSO
S+G
G+N
N+S
S+G+N
e.
NL eyes
p = 0.010
p = 0.048
-30 D eyes
p = 0.039
Change in axial length (mm)
DMSO 4+G2 G2+CP CP+4
f.
p = 0.008
Change in axial length (mm)
NL -30D
DMSO
4+G2
G2+CP
CP+4
g.
NL eyes
p = 0.006
-30 D eyes
p = 0.011
Changes in Refraction (D)
DMSO 4+G2 G2+CP CP+4
h.
p = 0.001
Changes in Refraction (D)
NL -30D
DMSO
4+G2
G2+CP
CP+4
i.
NL eyes
p = 0.00044
p = 0.00031
-30 D eyes
p = 0.00023
Change in VCD + RT (mm)
DMSO 4+G2 G2+CP CP+4
j.
p = 0.00042
Change in VCD + RT (mm)
NL -30D
DMSO
4+G2
G2+CP
CP+4

Supplemental Figure 7 | Effect of combined UPR inhibitor instillation on myopia development.

(a) Comparison of axial elongation between non-lens-wearing eyes (NL) and minus-lens-wearing eyes (-30D) in DMSO (as a control), STF080310 with GSK2656157 (S+G; 100 μM, respectively), GSK2656157 with nelfinavir (G+N; 100 μM, respectively), nelfinavir with STF080310 (N+S; 100 μM, respectively) and all inhibitors (S+G+N)-administrated mouse. The p values were determined by Student's two-tailed t-test. **(b)** Comparison of refraction between NL and -30D eyes in DMSO, S+G, G+N, N+S and S+G+N-administrated mouse. The p values were determined by Student's two-tailed t-test. **(c)** Effects of combined STF, GSK, and NFV eye drops on VCD+RT in NL and -30 D eyes (n = 5 per group). The p values were determined by two-tailed Generalized Estimating Equations. **(d)** Comparison of VCD+RT between NL and -30D eyes in DMSO, S+G, G+N, N+S and S+G+N-administrated mouse. The p values were determined by Student's two-tailed t-test. **(e)** Effects of combined 4μ8C, GSK2606414, and Ceapin A-7 instillation (4μ8C with GSK2606414: 4+G2, GSK2606414 with Ceapin A-7: G2+CP, Ceapin A-7 with 4μ8C: CP+4) eye drops on axial elongation in NL and -30 D eyes (n = 5 per group). The p values were determined by one-way ANOVA with Tukey HSD. **(f)** Comparison of axial elongation between NL and -30D eyes in DMSO, 4+G2, G2+CP and CP+4-administrated mouse. The p values were determined by Student's two-tailed t-test. **(g)** Effects of 4+G2, G2+CP and CP+4 instillation eye drops on refractive error in NL and -30 D eyes (n = 5 per group). The p values were determined by one-way ANOVA with Tukey HSD. **(h)** Comparison of refractive error between NL and -30D eyes in DMSO, 4+G2, G2+CP and CP+4-administrated mouse. The p values were determined by Student's two-tailed t-test. **(i)** Effects of 4+G2, G2+CP and CP+4 instillation eye drops

on VCD+RT in NL and -30 D eyes (n = 5 per group). The p values were determined by one-way ANOVA with Tukey HSD. **(j)** Comparison of VCD+RT between NL and -30D eyes in DMSO, 4+G2, G2+CP and CP+4-administrated mouse. The p values were determined by Student's two-tailed t-test. Source data are provided as a Source Data file.

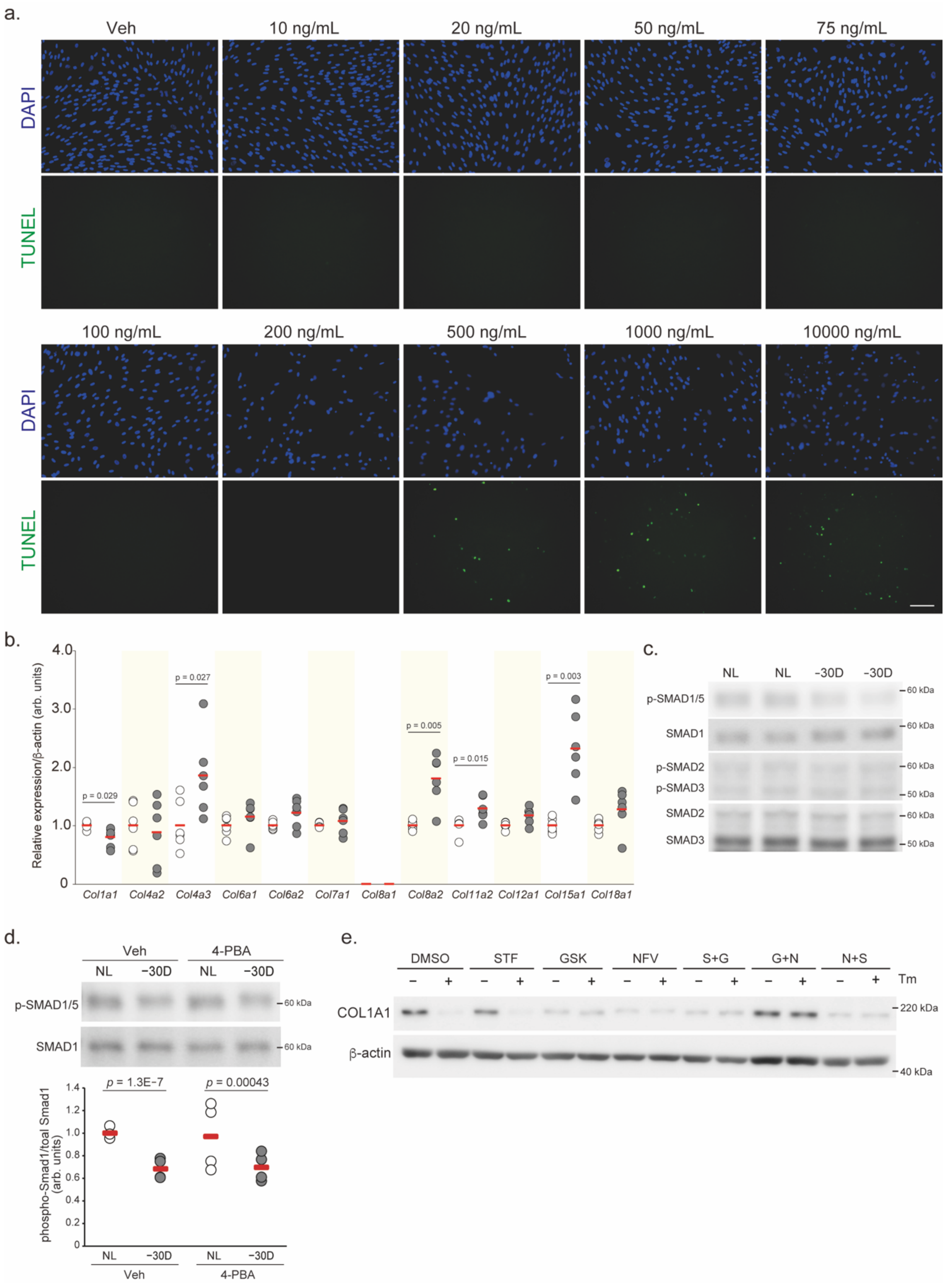
a.
Veh
10 ng/mL
20 ng/mL
50 ng/mL
75 ng/mL
DAPI
TUNEL
100 ng/mL
200 ng/mL
500 ng/mL
1000 ng/mL
10000 ng/mL
b.
Relative expression/β-actin (arb. units)
p = 0.029
p = 0.027
p = 0.005
p = 0.015
p = 0.003
Col1a1
Col4a2
Col4a3
Col6a1
Col6a2
Col7a1
Col8a1
Col8a2
Col11a2
Col12a1
Col15a1
Col18a1
c.
NL
NL
-30D
-30D
p-SMAD1/5
SMAD1
p-SMAD2
p-SMAD3
SMAD2
SMAD3
60 kDa
50 kDa
d.
Veh
4-PBA
NL
−30D
p-SMAD1/5
SMAD1
60 kDa
p = 1.3E−7
p = 0.00043
phospho-Smad1/toal Smad1 (arb. units)
e.
DMSO
STF
GSK
NFV
S+G
G+N
N+S
Tm
COL1A1
β-actin
220 kDa
40 kDa

Supplemental Figure 8 | Tunicamycin-induced dysregulation of collagen gene expression and its possible mechanism

(a) Effect of different concentrations of tunicamycin (Tm; 24 h) on cell death in human scleral fibroblasts. Upper panels: DAPI staining (blue), lower panel: TUNEL staining (green). Representative images of three biologically independent samples. **(b)** Effect of Tm (200 ng/mL) treatment on the expression of 12 LIM-sensitive collagen genes in human scleral fibroblasts (n = 6 per group). The p values were determined by Student's two-tailed t-test. **(c)** Immunoblots showing effect of minus lens-wearing on phosphorylation levels of SMAD1/5, SMAD2 and SMAD3. Representative blots from three independent experiments are shown. **(d)** Effect of LIM and 4-PBA instillation on phosphorylation of SMAD1. The p values were determined by two-tailed Generalized Estimating Equations. **(e)** Effect of IRE1, PERK and ATF6 inhibitors on the degradation of COL1A1 induced by tunicamycin (Tm, 200 ng/ml for 6 hours). Source data are provided as a Source Data file.

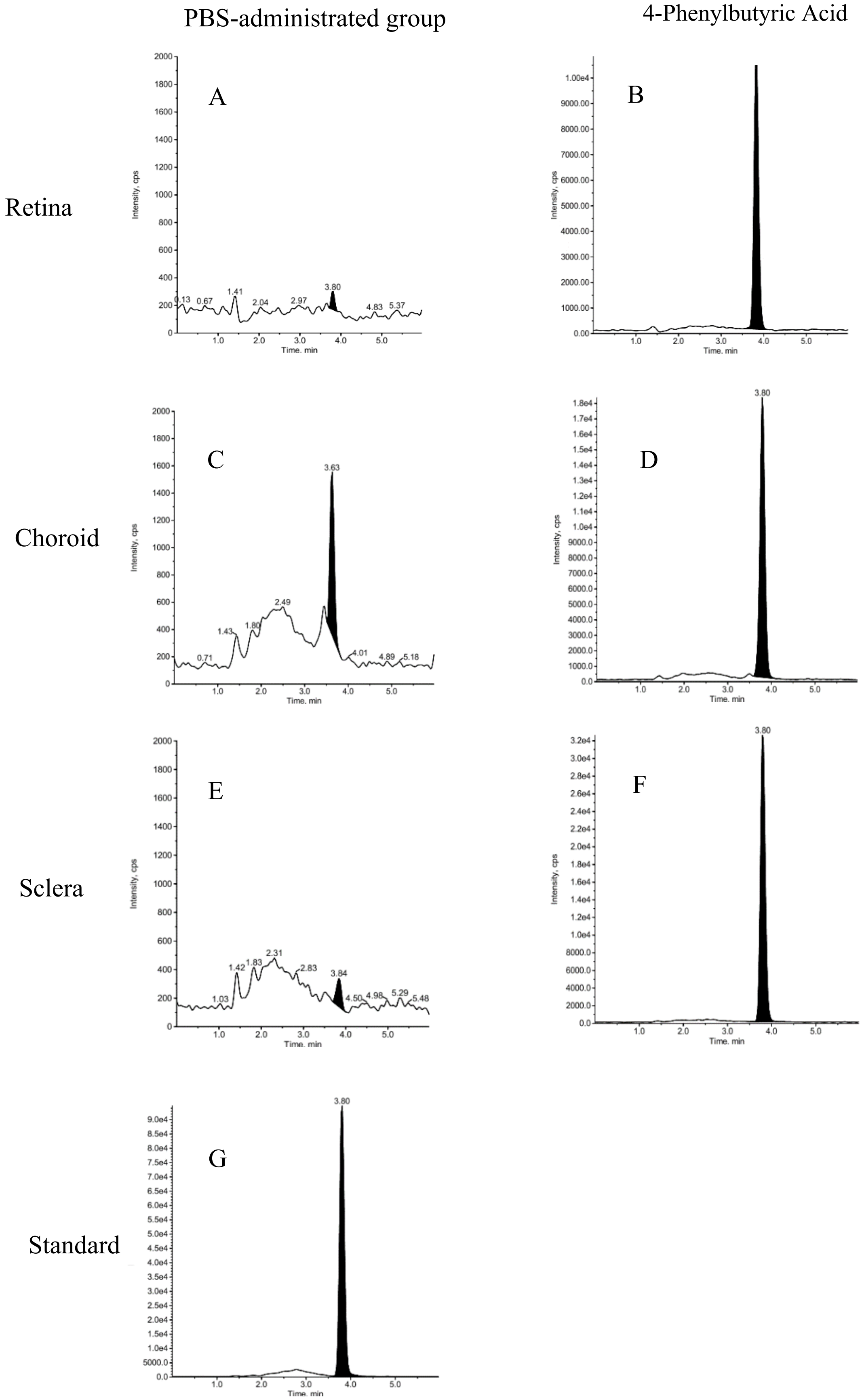
PBS-administrated group
4-Phenylbutyric Acid
Retina
Choroid
Sclera
Standard
A
B
C
D
E
F
G
Intensity, cps
Time, min
0.13
0.67
1.41
2.04
2.97
3.80
4.83
5.37
0.71
1.43
1.80
2.49
3.63
4.01
4.89
5.18
1.03
1.42
1.83
2.31
2.83
3.84
4.50
4.98
5.29
5.48

Supplemental Figure 9 | Representative chromatogram of 4-Phenylbutyric Acid (4-PBA) in mouse ocular tissues after 4-PBA or PBS treatment

A to F are chromatograms corresponding to the results in Table 4. As an example, the retention time of the peak detected with the standard reagent (2,000 ng/g) of 4-PBA is shown in G. Concentrations of 4-Phenylbutyric Acid were determined by the internal standard method. 0.57 mg of 4-Phenylbutyric Acid-d_{11} was weighed and dissolved in 5.7 mL of methanol to prepare an internal standard stock solution of 100 μg/mL. The prepared internal standard stock solution was diluted with methanol to prepare a 100 ng/mL internal standard working solution.

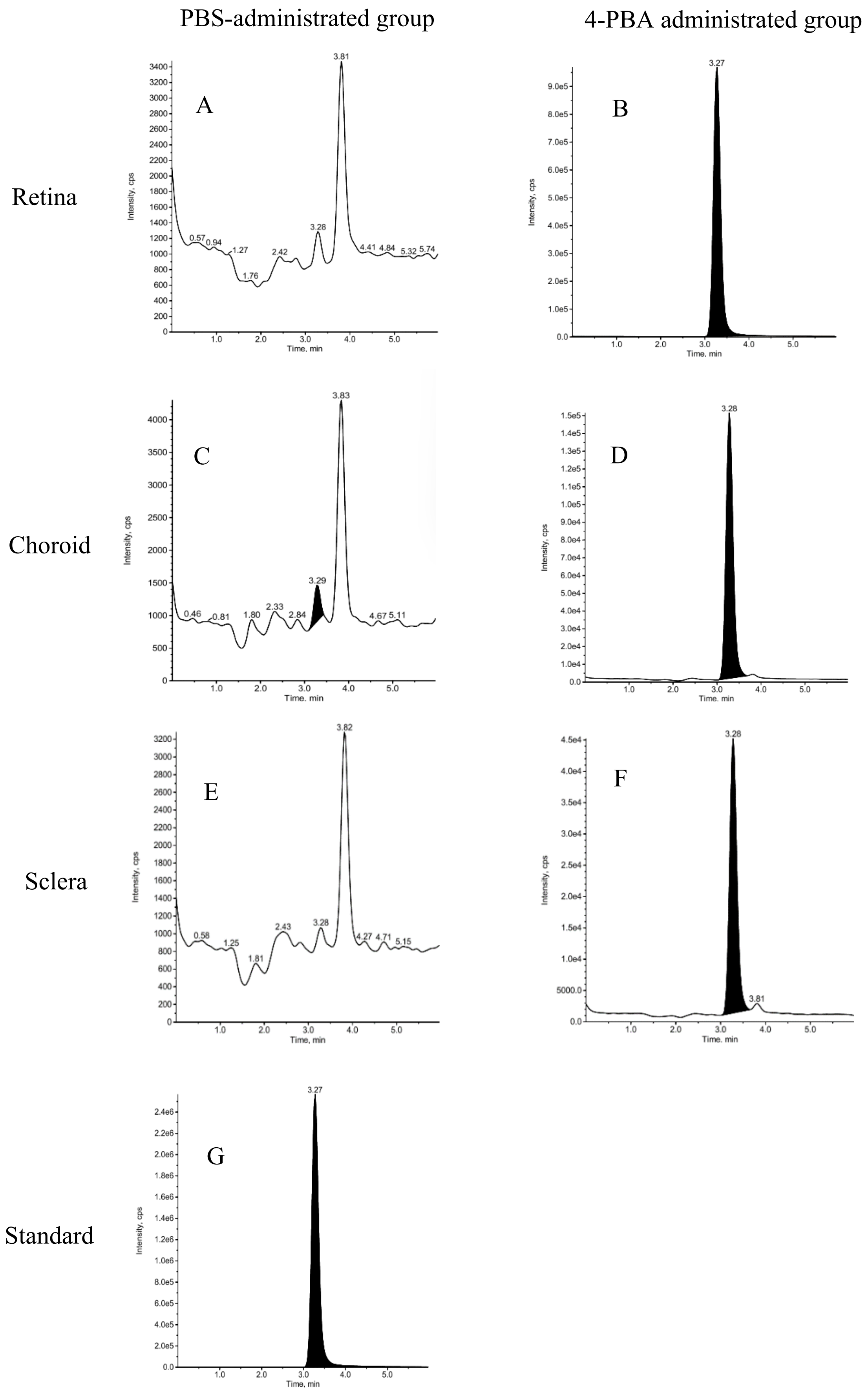
PBS-administrated group
4-PBA administrated group
Retina
Choroid
Sclera
Standard
A
B
C
D
E
F
G
Intensity, cps
Time, min
3.81
3.28
3.27
3.83
3.29
3.82

Supplemental Figure 10 | Representative chromatogram of Phenylacetic Acid (PAA) in mouse ocular tissues after 4-Phenylbutyric Acid or PBS treatment.

A to F are chromatograms corresponding to the results in Table 5. As an example, the retention time of the peak detected with the standard reagent (100,000 ng/g) of Phenylacetic Acid is shown in G. PAA was also determined by the internal standard method using 4-Phenylbutyric Acid-d_{11} as the internal standard reagent in the same way as 4-Phenylbutyric Acid. In the case of detection by LC/MS/MS, monitor ions were set for 4-Phenylbutyric Acid, PAA, and 4-Phenylbutyric Acid-d11, respectively, and detection was performed from the chromatograms obtained. Monitor ion are shown in the methods in the text.

gene	Forward primer(5'→3')	Reverse Primer(5'→3')
CHOP	ATATCTCATCCCCAGGAAACG	TCTTCCTTGCTCTTCCTCCTC
ATF4	CGAGATGAGCTTCCTG	GGAAAAGGCATCCTCC
GADD34	GAGGGACGCCCACAACTTC	TTACCAGAGACAGGGGTAGGT
SEL1L	GGTGTGCCACAACCTATGACT	TGGCTCTTCCTATTGCTTCCA
EDEM	GGGGCATGTTCGTCTTCGG	CGGCAGTAGATGGGGTTGAG
ERdj4	CTCCACAGTCAGTTTTCGTCTT	GGCCTTTTTGATTTGTCGCTC
ERdj5	GGAGCTGTCAACTGTGGTGAT	CCGATCTCCATTGTACTTCACTG
GRP78	TGTGGTACCCACCAAGAAGTC	TTCAGCTGTCACTCGGAGAAT
GRP94	CTCAGAAGACGCAGAAGACTCA	AAAACTTCACATTCCCTCTCCA
ERdj3	CCGGGACGCTTCCAAATGA	GGTACTCCATGCCATCTCGC
Eif2ak3	GACTGCGGAGACAACAGTGA	GACCGGGTATAGGGAGAAGC
Atf6	AAGCAGCTCAACACGAGGAT	CCTGTAGGAGAGGCATCAGC
Col1a1	GCTCCTCTTAGGGGCCACT	CCACGTCTCACCATTGGGG
Col1a2	AAGGGTGCTACTGGACTCCC	TTGTTACCGGATTCTCCTTTGG
Col2a1	GGGAATGTCCTCTGCGATGAC	GAAGGGGATCTCGGGGTTG
Col3a1	CTGTAACATGGAAACTGGGGAAA	CCATAGCTGAACTGAAAACCACC
Col4a1	CTGGCACAAAAGGGACGAG	ACGTGGCCGAGAATTTCACC
Col4a2	GACCGAGTGCGGTTCAAAG	CGCAGGGCACATCCAACTT
Col4a3	GGTGGGAAAATGTGATCCTGG	GCCTACGGATGGTTCTCCCT
Col4a4	ATGAGGTGCTTTTTCAGATGGAC	GGGGCCGCCATACTTCTTG
Col4a5	TTCCAGGTTTAGAAGGTCATCCA	ACGTTCTCCCTTGGTTCCATT
Col4a6	ATCGGATACTCCTTCCTCATGC	CCAGGGGAGACTAGGGACTG
Col5a1	TGAGTCTGGTTTTCCCGAGGA	GCCCTGCTCATTGTAAATGGAGA
Col5a2	ACAGGTGAAGTGGGATTCTCA	CCATAGCACCCATTGGACCA
Col5a3	CGGGGTACTCCTGGTCCTAC	GCATCCCTACTTCCCCCTTG
Col6a1	CTGCTGCTACAAGCCTGCT	CCCCATAAGGTTTCAGCCTCA
Col6a2	AAGGCCCCATTGGATTCCC	CTCCCTTCCGACCATCCGAT
Col6a3	GCTGCGGAATCACTTTGTGC	CACCTTGACACCTTTCTGGGT
Col6a4	CAGTACAGCGATACACCCACT	AGTCCACGGATGTGTTTCAAC
Col6a5	TGGGGCATCTAATGTTGGGG	CTCCAAGCGCAGATCCAGTG
Col7a1	GCCCAGAGATAGAGTGACCTG	CGCACTTCTCGAAAGTTGCTG
Col8a1	ACTCTGTCAGACTCATTCAGGC	CAAAGGCATGTGAGGGACTTG
Col8a2	GCCCTGGATGAGACTGGTATC	CGGTCAAACCTAACCGGCAT
Col9a1	CGACCGACCAGCACATCAA	AGGGGGACCCTTAATGCCT
Col9a2	AAGGGGCCTCCAGGTAAAGTT	TCCCATTAAACCATCAATGCCA
Col9a3	GGAATGCCGGGGTTCAAGG	AGTCCTCTTAATCCTCGTGGG
Col10a1	GGGACCCCAAGGACCTAAAG	GCCCAACTAGACCTATCTCACCT
Col11a1	AGGTGGAAAACGAAACGGTG	GGAAGAGAAAAGTCAAGGCGA
Col11a2	GAAGGGTGCTCGTGGGAAA	GAGGGCCTGGGTATCCTAGAG
Col12a1	AAGTTGACCCACCTTCCGAC	GGTCCACTGTTATTCTGTAACCC
Col13a1	GGAGCACCTGGACTAGACG	GCCTTGGACTGGTAAGCCAT
Col14a1	TTTGGCGGCTGCTTGTTTC	CGCTTTTGTTGCAGTGTTCTG
Col15a1	GCCAGCGGGTTATCCTCTAC	ATCACGGGCACGGAGAAAAC
Col16a1	GAGAGCGAGGATACACTGGC	CTGGCCTTGAAATCCCTGG
Col17a1	AAGTCACCGAGAGAATTGTCAC	AGAGAGCCTGTCTTAGCATATCC
Col18a1	GTGCCCATCGTCAACCTGAA	GACATCTCTGCCGTCAAAAGAA
Col19a1	GGCTCTTGGAAATTGTGGACC	ACTTCCCAACTTGAAACAGGTT
Col20a1	AGCCGACTCATTTGCCAAAAA	GGGTGGGTATAAGGCTGGAG
Col22a1	GGGGAACCTGGATACGCTAAA	CAAAGTACGCACACTGGGAG
Col23a1	CCCCATCTGAGTGCATCTGTC	CTTGCCGTCCAGACCTAGAG
Col24a1	TTCACTGTCTAAACACCCCAAGG	CCATCCTGAATCTTGCAGTCAT
Col25a1	GGGGTCAAAGGGTGATCGTG	CCCGTGACAGTTAAGGTGGTAA
Col26a1	AGAGGGGAGTTTGGGACTGT	GGACCAGTAGGCTCAGCAAG
Col27a1	CCTTCCCGTAGGGACTCCAT	GGCACAGTAATTGTGAGCGAC
Col28a1	ACCCGTTCTACTATTGAGTGACC	GCTCGCCCTTTTCACATTCA
Muc1	TCTCCAGCCACCAGCCCTCTAA	TGGCCATGGTAGGAGAAACAGG
Muc4	CTCCAAGAAATGTAGTGGCTTTCA	CACGGTCTTGGGCTGGAGTA
Muc5ac	AAAGACACCAGTAGTCACTCAGCAA	CTGGGAAGTCAGTGTCAAACCA
β-actin	GGAGGAAGAGGATGCGGCA	GAAGCTGTGCTATGTTGCTCTA
Hprt	TCAGTCAACGGGGGACATAAA	GGGGCTGTACTGCTTAACCAG

Supplemental Table 1 | List of Primers (mouse) used in present study.

gene	Forward primer(5'→3')	Reverse Primer(5'→3')
chick CHOP	AGCTGAGTGCACACAACGAG	GCTGTACAGTGGTGCTGGAA
chick GRP78	CCTGGAATGACCCCTCTGTA	CTTGCCCAGATAAGCCTCTG
chick β-actin	GCGCTCGTTGTTGACAAT	CATCACCAACGTAGCTGTCTTT

Supplemental Table 2 | List of Primers (chick) used in present study.

gene	Forward primer(5'→3')	Reverse Primer(5'→3')
human Col1a1	GAGGGCCAAGACGAAGACATC	CAGATCACGTCATCGCACAAC
human Col4a2	AAGGGCTTCATCGGAGACC	CCAGCGTCACCTTTCCACC
human Col4a3	GCAGTGGTTCTAAGGGTGAGC	GAAAGCCAAAGAATCCCGGAG
human Col6a1	ACAGTGACGAGGTGGAGATCA	GATAGCGCAGTCGGTGTAGG
human Col6a2	GACTCCACCGAGATCGACCA	CTTGTAGCACTCTCCGTAGGC
human Col7a1	TTACGCCGCTGACATTGTGTT	ACCAGCCCTTCGAGAAAGC
human Col8a1	GGGAGTGCTGCTTACCATTTC	AGCGGCTTGATCCCATAGTAG
human Col8a2	GCTGGCTTAGGCAAACCTG	GGACTCCCACACCGTCTACT
human Col11a2	GCTCCCCTCCTGACTCTCTAC	CCGGGTGACTCGCTTCTTG
human Col12a1	AGCTGAGGCAGACATTGTGTT	CCTCCTTTGTACGGCAAGTTT
human Col15a1	CTGCCCTCGTCCGTATCCT	CTGATGGCGAAGTCCCTGA
human Col18a1	CAGTGGACACACTTAGCCCTC	GCGGCATTCTCTGGAACTCC
human β-actin	CACCATTGGCAATGAGCGGTTC	AGGTCTTTGCGGATGTCCACGT

Supplemental Table 3 | List of Primers (human) used in present study.

Treatment	Sample	Concentration of 4-Phenylbutyric Acid (ng/g)				
		1	2	3	Mean	SD
4-Phenylbutyric Acid	Retina	209	221	165	198	29
	Choroid	327	520	208	352	157
	Sclera	829	691	117	546	378
PBS	Retina	BLQ	BLQ	BLQ	BLQ	NC
	Choroid	BLQ	BLQ	BLQ	BLQ	NC
	Sclera	BLQ	BLQ	BLQ	BLQ	NC

BLQ : Below the lower limit of quantification

Supplemental Table 4 | Concentrations of 4-PBA in mouse ocular tissues after 4-PBA treatment.

Treatment	Sample	Concentration of Phenylacetic Acid (ng/g)				
		1	2	3	Mean	SD
4-Phenylbutyric Acid	Retina	34100	33900	30900	33000	1800
	Choroid	7780	5120	6040	6310	1350
	Sclera	1610	1480	1500	1530	70
PBS	Retina	BLQ	BLQ	BLQ	BLQ	NC
	Choroid	BLQ	BLQ	BLQ	BLQ	NC
	Sclera	BLQ	BLQ	BLQ	BLQ	NC

BLQ : Below the lower limit of quantification
NC: Not calculated

Supplemental Table 5 | Concentrations of Phenylacetic acid in mouse ocular tissues after 4-PBA treatment.

Treatment	Sample		Concentration of 4-Phenylbutyric Acid (ng/g or ng/mL)			
			Retina (right)	Choroid	Sclera	Plasma
4-Phenylbutyric Acid	1	-	BLQ	BLQ	BLQ	BLQ
	2	15 min	349	348	203	394
	3	30 min	88.2	117	107	186
	4	60 min	27.9	76.7	44.9	76.2

BLQ : Below the lower limit of quantification

Supplemental Table 6 | Concentrations of 4-PBA in mouse ocular tissues and plasma after 4-PBA ocular administration.

Treatment	Sample		Concentration of Phenylacetic Acid (ng/g or ng/mL)			
			Retina (right)	Choroid	Sclera	Plasma
4-Phenylbutyric Acid	1	-	15.4	16.1	22.0	61.4
	2	15 min	51.1	66.7	96.3	87.5
	3	30 min	50.2	49.2	38.8	69.8
	4	60 min	21.6	29.5	48.3	38.3

BLQ : Below the lower limit of quantification

Supplemental Table 7 | Concentrations of Phenylacetic Acid in mouse ocular tissues and plasma after 4-PBA ocular administration.

Agonists/Antagonists	Abbreviations in this paper	Action and mechanism
4-phenylbutyric Acid	4-PBA	Acts as a chemical chaperone to aid in protein folding and consequently reduce ER stress
Tauroursodeoxycholic Acid	TUDCA	Chemical chaperone which can inhibit unfold protein response dysfunction and ameliorate ER stress
Tunicamycin	Tm	Inhibits glycoprotein biosynthesis in the ER which result in accumulation of misfolding protein, follwed by induction of ER stress
Thapsigargin	TG	Inhibitis sarco/endoplasmic reticulum Ca2+-ATPase which caruse ER stress
STF083010	STF or S	IRE1 inhibitor through blocking IRE1 endonuclease activity
4μ8C	4μ8C or 4	IRE1 inhibitor through blocking substrate access to the active site of IRE1
GSK2656157	GSK or G	PERK inhibitor thorogh competing with ATP
GSK2606414	GSK2606414 or G2	The first PERK inhibitors
Nerfinavir	NFV or N	ATF6 inhibitor through blocking Site-2 proteinase cleavage of ATF6
Ceapin-7	Ceapin or CP	ATF6 inhibitor through preventing transport of ATF6a to the Golgi apparatus
CCT020312	CCT	A selective PERK activator which elicits eIF2 phosphorylation
AA147	AA	A selective ATF6 activator

Supplemental Table 8 | Compounds for intervention in ER stress used in this study.
