# Supplementary material for: Scleral PERK and ATF6 as targets of myopic axial elongation of mouse eyes
Source: Nat Commun. 2022 Oct 10;13:5859. doi: 10.1038/s41467-022-33605-1 (PMC9550863; doi:10.1038/s41467-022-33605-1)
Supplement: Supplementary file 2 — Reporting Summary [file 41467_2022_33605_MOESM2_ESM.pdf]

nature portfolio

Corresponding author(s): Toshihide Kurihara, Kazuo Tsubota

Last updated by author(s): Mar 7, 2022

Reporting Summary

Nature Portfolio wishes to improve the reproducibility of the work that we publish. This form provides structure for consistency and transparency in reporting. For further information on Nature Portfolio policies, see our Editorial Policies and the Editorial Policy Checklist.

Statistics

For all statistical analyses, confirm that the following items are present in the figure legend, table legend, main text, or Methods section.

n/a	Confirmed	
☐	☒	The exact sample size (n) for each experimental group/condition, given as a discrete number and unit of measurement
☐	☒	A statement on whether measurements were taken from distinct samples or whether the same sample was measured repeatedly
☐	☒	The statistical test(s) used AND whether they are one- or two-sided<br>*Only common tests should be described solely by name; describe more complex techniques in the Methods section.*
☐	☒	A description of all covariates tested
☐	☒	A description of any assumptions or corrections, such as tests of normality and adjustment for multiple comparisons
☐	☒	A full description of the statistical parameters including central tendency (e.g. means) or other basic estimates (e.g. regression coefficient) AND variation (e.g. standard deviation) or associated estimates of uncertainty (e.g. confidence intervals)
☐	☒	For null hypothesis testing, the test statistic (e.g. F, t, r) with confidence intervals, effect sizes, degrees of freedom and P value noted<br>*Give P values as exact values whenever suitable.*
☒	☐	For Bayesian analysis, information on the choice of priors and Markov chain Monte Carlo settings
☒	☐	For hierarchical and complex designs, identification of the appropriate level for tests and full reporting of outcomes
☒	☐	Estimates of effect sizes (e.g. Cohen's d, Pearson's r), indicating how they were calculated

Our web collection on statistics for biologists contains articles on many of the points above.

Software and code

Policy information about availability of computer code

Data collection	No software was used for data collection.
Data analysis	SPSS statistics27, G*Power software ver 3.1.9, ImageJ 1.53 software

For manuscripts utilizing custom algorithms or software that are central to the research but not yet described in published literature, software must be made available to editors and reviewers. We strongly encourage code deposition in a community repository (e.g. GitHub). See the Nature Portfolio guidelines for submitting code & software for further information.

Data

Policy information about availability of data

All manuscripts must include a data availability statement. This statement should provide the following information, where applicable:

- Accession codes, unique identifiers, or web links for publicly available datasets
- A description of any restrictions on data availability
- For clinical datasets or third party data, please ensure that the statement adheres to our policy

The all data are available within the manuscript as Supplementary Information or Source Data file. Source data are provided with this manuscript.

Field-specific reporting

Life sciences study design

All studies must disclose on these points even when the disclosure is negative.

Sample size	A preliminary LIM experiment was conducted (no lens control vs minus lens wearing: n=6) and the sample size was calculated by G*power software based on the mean and SD values.
Data exclusions	A certain number of mice have corneas that are not flat or have been damaged during the breeding process, which makes it impossible to accurately measure axial length and refraction.For the reasons mentioned above, a cloudy cornea is difficult for light to pass through and is considered to be a different light environment than a normal individual. Since the light environment is a major factor in the myopia induction experiments in this study, we excluded individuals with this condition from our analysis.
Replication	Measurements of ocular axial length and refraction, histological experiment (TEM, IHC, TUNEL, scleral falt mount), Western blotting, and qPCR were performed at least two independent experiments to ensure that similar results were obtained and all replications had same tendency.
Randomization	Purchased mice were randomly assigned to cages by a laboratory assistant not included in the authors of this paper, who randomly determined the treatment for each cage. In cell culture experiments, the plates and wells used for the experimental treatment were randomly selected from several that were sown with the same number of cells.
Blinding	When measuring axial length and refraction, the person administering the chemicals to the mice and the person taking the measurements were separated, and the person taking the measurements did not know which treatment the mice had received. After sample preparation, blinding was basically not carried out because we had to understand the relationship between the sample and the treatment.

Reporting for specific materials, systems and methods

We require information from authors about some types of materials, experimental systems and methods used in many studies. Here, indicate whether each material, system or method listed is relevant to your study. If you are not sure if a list item applies to your research, read the appropriate section before selecting a response.

Materials & experimental systems

n/a	Involved in the study	
☐	☒	Antibodies
☐	☒	Eukaryotic cell lines
☒	☐	Palaeontology and archaeology
☐	☒	Animals and other organisms
☒	☐	Human research participants
☒	☐	Clinical data
☒	☐	Dual use research of concern

Methods

n/a	Involved in the study	
☒	☐	ChIP-seq
☒	☐	Flow cytometry
☒	☐	MRI-based neuroimaging

Antibodies

Antibodies used	anti-ATF6 (1:1000,73-505, Bio Academina, Osaka, Japan); IRE1 alpha (phosphor Ser724) antibody (1:1000, GTX132808, GeneTex, CA, USA); IRE1 (14C10) Rabbit mAb (1:1000, #3294, Cell Signaling Technologies Japan, Tokyo, Japan); phosphor-eIF2α (Ser51) (D9G8) XP Rabbit mAb (1:1000, #3398, Cell Signaling Technologies Japan, Tokyo, Japan); eIF2α (D7D3) XP Rabbit mAb (1:1000, #5324, Cell Signaling Technologies Japan, Tokyo, Japan); phospho-Smad1/5 (1:1000, Ser463/465) (41D10) rabbit mAb (1:1000, #9516, Cell Signaling Technologies Japan, Tokyo, Japan); Smad1 (D59D7) XP Rabbit mAb (1:1000, #6944, Cell Signaling Technologies Japan, Tokyo, Japan); phosphor-Smad2 (S465/467)/Smad3 (S423/425)(D27F4) Rabbit mAb(1:1000, #8828, Cell Signaling Technologies Japan, Tokyo, Japan); Smad2/3 (D7G7) XP Rabbit mAb (1:1000, #8685, Cell Signaling Technologies Japan, Tokyo, Japan); β-actin (8H10D10) Mouse mAb (1:5000, #3700, Cell Signaling Technologies Japan, Tokyo, Japan); BiP (C50B12) Rabbit mAb (1:100, #3177, Cell Signaling Technologies Japan); Alexa Fluor 555-conjugated anti-rabbit IgG (1:200, A-21428, Life Technology Japan, Tokyo, Japan) , Anti-rabbit IgG, HRP-linked Antibody (1:10000, #7074, Cell Signaling Technologies Japan, Tokyo, Japan), Anti-mouse IgG, HRP-linked Antibody (1:10000, #7076, Cell Signaling Technologies Japan, Tokyo, Japan)
Validation	Westen blotting of tunicamycin-treated human fibroblasts with lysate shows a band whose expression level is enhanced compared to that of untreated cells and is around the estimated molecular weight (ATF6, p-eIF2, eIF2, pIRE1 and IRE1 were validated by both Tm-treated experiments and molecular weight. All SMAD were validated by only molecular weight). BiP antibody for IHC was verified by comparison with 1st antibody-negative slides. All antibodies were also validated for each applications on manufacturer's website or data sheets.

Eukaryotic cell lines

Policy information about cell lines

Cell line source(s)	Primary human scleral fibroblasts were directly purchased from Lifeline Cell Technology (Frederick, MD, USA).
Authentication	The cells used in this study were directly purchased as an authenticated product and we did not perform the Cell Line Authentication ourselves.
Mycoplasma contamination	Done
Commonly misidentified lines (See ICLAC register)	Non

Animals and other organisms

Policy information about studies involving animals; ARRIVE guidelines recommended for reporting animal research

Laboratory animals	Mouse (C57BL6J, male, 3-week-old, 24±2 °C and 40-60 %), White Leghorn chicks (Male, 5-days-old)
Wild animals	No wild animals were used in this study.
Field-collected samples	No filed-collected samples were used in this study.
Ethics oversight	All animal experiments in this study were approved by the Animal Experimental Committee of Keio University and adhered to the Institutional Guidelines on Animal Experimentation at Keio University, the ARVO Statement for the Use of Animals in Ophthalmic and Vision Research, and the Animal Research: Reporting of In Vivo Experiments (ARRIVE) guidelines for the use of animals in research.

Note that full information on the approval of the study protocol must also be provided in the manuscript.
